# NMDA Neurotransmission Dysfunction in Behavioral and Psychological Symptoms of Alzheimer’s Disease

**DOI:** 10.2174/157015912803217288

**Published:** 2012-09

**Authors:** Yu-Jhen Huang, Chieh-Hsin Lin, Hsien-Yuan Lane, Guochuan E Tsai

**Affiliations:** a Institute of Clinical Medical Science, China Medical University, Taichung, Taiwan; b Department of Psychiatry, China Medical University Hospital, Taichung, Taiwan; c Department of Psychiatry, Chang Gung Memorial Hospital – Kaohsiung Medical Center, Chang Gung University College of Medicine, Kaohsiung, Taiwan; d Department of Psychiatry, Harbor-UCLA Medical Center, Torrance, California, and Los Angeles Biomedical Research Institute

**Keywords:** D-serine, glycine transporter, memantine, sarcosine.

## Abstract

Dementia has become an all-important disease because the population is aging rapidly and the cost of health care associated with dementia is ever increasing. In addition to cognitive function impairment, associated behavioral and psychological symptoms of dementia (BPSD) worsen patient’s quality of life and increase caregiver’s burden. Alzheimer’s disease is the most common type of dementia and both behavioral disturbance and cognitive impairment of Alzheimer’s disease are thought to be associated with the N-methyl-D-aspartate (NMDA) dysfunction as increasing evidence of dysfunctional glutamatergic neurotransmission had been reported in behavioral changes and cognitive decline in Alzheimer’s disease. We review the literature regarding dementia (especially Alzheimer’s disease), BPSD and relevant findings on glutamatergic and NMDA neurotransmission, including the effects of memantine, a NMDA receptor antagonist, and NMDA-enhancing agents, such as D-serine and D-cycloserine. Literatures suggest that behavioral disturbance and cognitive impairment of Alzheimer’s disease may be associated with excitatory neurotoxic effects which result in impairment of neuronal plasticity and degenerative processes. Memantine shows benefits in improving cognition, function, agitation/aggression and delusion in Alzheimer’s disease. On the other hand, some NMDA modulators which enhance NMDA function through the co-agonist binding site can also improve cognitive function and psychotic symptoms. We propose that modulating NMDA neurotransmission is effective in treating behavioral and psychological symptoms of Alzheimer’s disease. Prospective study using NMDA enhancers in patients with Alzheimer’s disease and associated behavioral disturbance is needed to verify this hypothesis.

## INTRODUCTION

Dementia is a devastating mental disorder with high morbidity and mortality [1]. The prevalence of dementia doubles every 5 years in the elderly people aged between 65 to 85 years old [2]. It affects 1.5% of the population at the age of 65 and >20% by the age of 85 [3]. The overall age structure also favors an ever-increasing rate of dementia due to the extension of life span. In 2005, up to 24.3 million people were suffering from dementia worldwide [4]. In 2010, it was estimated that 35 million people in the world had dementia [5]. The population of dementia is expected to reach 65 million by 2030 and 113 million by 2050 [5]. The high prevalence and incidence of dementia in the elderly are noteworthy health and social problems. Nowadays, the underlying total cost of health care for dementia patients is as high as $385 billion [1] , which representing a heavy care burden to families of such patients and whole society.

### NMDA Neurotransmission Plays a Vital Role in Cognitive Function

Dementia is a disorder of memory and cognitive impairment. The most common type of dementia is Alzheimer’s disease (AD), which is a neurodegenerative disorder. Many neurotransmitter systems are involved in the pathophysiology of Alzheimer’s disease and a critical one of them is N-Methyl-D-aspartate receptor (NMDAR)-mediated neurotransmission [6, 7]. 

The NMDA receptor (NMDAR) is a tetramer composed of two NR1 subunits and two NR2 subunits or less commonly, two NR3 subunits. NMDAR activation leads to Ca^2+^ influx and triggers downstream signal transduction. Once NMDAR is activated, Ca^2+^ enters the cell. Then, calmodulin-dependent kinase (CaMK) and cAMP response element-binding (CREB) protein are phosphorylated and trigger gene transcription needed for long term potentiation (LTP) formation. This is the molecular basis of neural plasticity and memory formation [8]. NMDA neurotransmission also activates protein kinase-A (PKA) and Ras pathway [9]. The former can in turn modulate NMDAR function [9] and the latter further induces mitogen-activated protein kinase (MAPK)/extracellular signal-regulated kinase (ERK) and phosphoinositide 3 (PI3K)/Akt/mammalian target of rapamycin (mTOR) pathways, which are important for neural survival [10].

Activation of NMDAR requires occupation of both glutamate and “glycine” binding sites. Although it was named “glycine” binding site, D-serine is also endogenous full agonist that is more or equally potent than glycine. The glutamate-binding site is on the NR2 subunit, and the glycine-binding site is on the NR1 subunit [11]. Each NR subunit has its own isoforms or subtypes. The NR1 subunit has 8 isoforms by alternative RNA splicing, and there are 4 subtypes of NR2 subunit and 2 subtypes of NR3 subunit from different encoding genes. Different compositions of NMDARs have different anatomical distributions, developmental profile and functional properties [12, 13]. The subunit composition and number of NMDARs in the synaptic surface are regulated by NMDAR trafficking, which is a dynamic process involving receptor retrieval, endocytosis, and lateral diffusion [14]. NMDAR trafficking can be regulated by synaptic activity. For example, LTP leads to expression of synaptic NMDAR [15] and long-term depression (LTD) is associated with NMDAR endocytosis [16]. Besides, glycogen synthase kinase 3 (GSK-3), a key kinase of signal transduction involved in neuroprotection, also regulates NMDAR trafficking. Inhibition of GSK-3 activity increases NMDAR internalization and reduces NMDAR-mediated current [17].

The dysregulation of NMDAR trafficking is associated with neuropsychiatric disorders, such as AD [14]. It is consistent with the critical role NMDA neurotransmission plays in the memory formation, learning and neuronal plasticity [18-20]. Increasing NMDAR function by over-expression or underdegradation of NR2B subunit in hippocampus can enhance LTP and learning [21, 22]. On the other hand, decrease of NR2B-containing receptor impairs LTP induction [20] and elimination of NR2B subunit function in area CA1 of hippocampus reduces neuron dendritic spine density and interferes with memory consolidation and learning [23, 24]. Specific ablation of NMDAR gene in the CA3 pyramidal cells of hippocampus in adult mice also results in deficits in their associative memory recall [25]. Besides, pharmacological blockade of NMDAR function leads to brain atrophy, impaired neuroplasticity, and learning disability [26-28]. 

### Bi-directional Involvement of NMDAR Function in the Pathophysiology of Alzheimer’s Disease

Although NMDAR function is vital for memory and cognitive function, its role in the pathophysiology of AD is still not completely understood. NMDAR over-activation by glutamate leads to cell death mediated by calcium overload, which is called excitotoxicity [6, 29]. The excitotoxicity is one of the accepted neurochemical model of AD. The underlying mechanism of excitotoxicity in pathophysiology of AD may be involved with amyloid-β peptide (Aβ), which is the hallmark of the pathogenesis of AD [30]. There are mutual interactions between NMDAR and Aβ. Aß increases NMDAR activity [31, 32], induces inward Ca^2+^ current and neurotoxicity [33]. In turn, NMDAR activation stimulates Aβ production [34-36] and Aβ associated synaptic loss may be NMDA–dependent [37].

On the contrary, the NMDA signaling pathways in the cerebral cortex and hippocampus are impaired in the aging brain [38]. Synaptic NMDA neurotransmission is crucial to neuronal survival. Synaptic NMDAR hypofunction leads to apoptosis [39, 40]. Blockade of NMDAR function by gene deletion of NMDAR or using NMDAR antagonist increases apoptotic cell death [39-45]. For example, MK-801, an NMDAR antagonist, activates caspase-3 pathway and triggers apoptotic pathway [46]. These complex neuroprotective/neurotoxic effects of NMDAR relies on many underlying signaling pathways, including ERK, Akt and GSK pathways [47]. NMDAR antagonists lead to apoptotic neurodegeneration through impairing ERK/CREB pathway and *Bcl2* expression [48]. Decrease of Akt activity and increase of GSK activity are found after NMDAR antagonist administration. Inhibition of GSK activity can decrease caspase-3 activity and block NMDAR antagonist-induced neurotoxicity [49]. This NMDAR hypoactivity-induced neurodegeneration is postulated to contribute to the pathogenesis of AD [50, 51]. 

Other studies also suggest that NMDAR hypofunction is related to brain dysfunction in aging. Decreased NMDAR activity by knocking down NR2B expression in young rats leads to impairment of LTP and spatial learning, which mimics age-related deficits [52]. The aging brain with declining memory and cognitive function is associated with decreased NMDAR [53, 54], change of NR subunit composition [55-57], diminished NMDAR binding activity [58] and attenuated NMDA-dependent LTP [59]. Besides, the redox site on NMDARs is in a more oxidized state in aged rats than that in young ones and this altered redox state may lead to reduced NMDR responses through Ca^2+^/CaMKII dependent mechanism during aging [60].

NMDAR hypofunction may be involved in the progression of aging brain from mild cognitive impairment to AD. Blockade of NMDAR function by NMDAR antagonist in rhesus monkey impairs visuo-spatial paired-associate learning, which represents early cognitive impairment of AD [61]. Individuals with AD or merely mild cognitive impairment have fewer NMDAR in the frontal cortex and hippocampus [62, 63]. One study of genetic polymorphisms of NR2B subunit promoter in sporadic AD suggests that allele which leads to lower NR2B subunit expression is associated with AD [64]. In the genetic mouse model of AD, expression of surface NMDAR in neuron is decreased [65] and NMDAR-mediated response is impaired progressively with age [66, 67]. In another study, NR1/NR2B receptor expression levels are reduced with increasing pathological severity in the post-mortem tissue of AD patients [68]. In addition to reduced number of NMDARs, disrupted glutamatergic neurotransmission [34], decreased CSF concentrations of excitatory amino acids [69], decreased serum level of D-serine [70] and reduced D-aspartate uptake [71] are also noted in AD.

Furthermore, the interactions between acetylcholine (ACh) and NMDA neurotransmission may account for the pathophysiology of AD. ACh is one of major neurotransmitter in central nervous system and is crucial for memory and cognition. The cholinergic hypothesis is one of the most acceptable mechanisms about pathophysiology of AD. Cognitive decline in aging and dementia are related to decreased cholinergic function [72, 73]. Administration of anticholinergic drugs results in memory impairments which resemble AD [74]. Loss of cholinergic neurons and decreased synaptic ACh level are found in the brain of AD [75] and the extent of cholinergic deficits correlates with the severity of AD [76]. Clinically, acetylcholinesterase (AChE) inhibitors which increase synaptic ACh level by decreasing degradation of ACh are one of the treatment options in AD [77]. 

Both upreguation and downregulartion are found in the interaction between ACh and NMDA neurotransmission. Some studies suggest that ACh can potentiate NMDAR related signaling pathways in the hippocampus [78, 79]. Other studies indicate that ACh and nicotinic ACh receptor agonist inhibit NMDAR-mediated currents [80]. Besides, AChE inhibitor may exert its neuroprotective effect by decreasing glutamate excitotocity. AChE inhibitor can reduce expression of NR1 subunit on cell surface, decrease glutamate-mediated Ca^2+^ influx [81] and reduce NMDAR mediated excitatory current through nicotinic receptor mediated Ca^2+^- dependent mechanism and activation of ERK pathway [82]. The modulation of AChE inhibitor on NMDA neurotransmission is impaired in amyloid precursor protein (APP) transgenic mouse model of AD [82].

As mentioned before, Aβ deposition plays an important role in pathophysiology of AD. Although some evidences show that the underlying mechanism of glutamate excitotocity in AD may be related to Aβ deposition, the opposing findings about the modulation between NMDA neurotransmission and Aβ formation are found. NMDAR activation can reduce Aβ production by enhancing non-amyloidogenic processing of APP [83, 84]. On the other hand, Aβ aggregation interferes with NMDA neurotransmission, which may lead to cognitive function impairment as Aβ impairs NMDA–dependent LTP both *in vivo* and *in vitro* [85-87]. Aβ also suppresses NMDAR-dependent synaptic function [88] by decreasing NR2 tyrosine phosphorylation, increasing endocytosis of NMDAR [65, 89, 90] and interfering with signal transduction of NMDAR, including the Ca^2+^-dependent protein phosphatase calcineurin [91], Ca^2+^/calmodulin-dependent protein kinase II [92], protein phosphatase [93], and CREB [91]. In addition to Aβ, apolipoprotein E4 (ApoE 4), an amyloid binding protein isoform related to the AD risk, impairs glutamatergic neurotransmission by reducing NMDAR function [94]. 

### Conceptualizing NMDA Modulation Treatment for Alzheimer’s Disease

Either over or under-function of NMDA neurotransmission brings forth cognitive dysfunction or neurotoxicity. A balanced NMDAR activity is required for optimal brain function. A recent model of this “NMDA paradox” indicates that it may be related to different composition of NR subunits and receptor localization. Synaptic NMDARs, mostly assembled by NR1 and NR2A subunits, trigger the signal transduction of cell survival pathways, whereas extrasynaptic NMDARs, composed of NR1 and NR2B subunits, involve with the signal pathways of cell death [10, 11]. This complexity implies that NMDAR activity needs to be kept in an optimal range for brain function. Accordingly, normalizing NMDAR dysfunction by enhancing NR1/NR2A while avoiding excitotoxicity due to NR1/NR2B could be a better approach instead of straight NMDAR antagonism, which will impair normal physiological function like memory and cognition, desperately needed in treatment of AD, and induce side effects like apoptosis and psychosis.

In 1990's, most major pharmaceutical companies rushed to develop NMDAR antagonists as neuroprotectants for AD under the basis of “glutamate excitotoxicity theory”. However, administration of NMDAR antagonists which block the receptor completely has limited usefulness clinically due to the severe side effects such as psychosis, nausea, vomiting, memory impairment, and neuronal cell death. Several large-scale Phase III trials of NMDAR antagonists fail to show neuroprotective effects with the exception of memantine [95]. In fact, many NMDAR antagonists, such as ketamine, MK801 cause cognitive impairment and psychosis [96-100]. Different from other NMDAR antagonists, memantine has been demonstrated to have beneficial effects for patients with moderate–to–severe AD, while not mild AD [101]. Memantine is an uncompetitive NMDAR weak partial antagonist of low affinity, which supposedly can block the NMDAR over-activation by preventing excessive influx of calcium without affecting physiological NMDAR activity [102-104]. Consistently, therapeutically relevant plasma concentration of memantine produces only 30% NMDAR occupancy [105].

Other NMDA modulators have been investigated for therapeutic effect on the treatment of AD. One way to modulate NMDAR function is to enhance the function through the co-agonist binding site. Augmentation through the NMDAR-glycine binding site is preferred to avoid the excitotoxicity mediated through the glutamate binding site [106]. Enhancers of NMDAR-glycine binding site include full agonist (*e.g.* glycine, D-serine and D-alanine), partial agonist (*e.g*. D-cycloserine), and glycine transporter 1 (GlyT1) inhibitor (*e.g.* sarcosine), that blocks the reuptake of glycine [107]. In mouse model, the learning deficits caused by NMDAR hypofunction in mice with point mutations in NMDAR glycine binding site can be rescued by administration of D-serine [108, 109]. Supporting neurotrophic/cognitive effects, D-cycloserine can improve cognitive functions in animal study [110-112]. The learning and memory enhancing effects of D-cycloserine are in a dose-dependent manner [110]. However, the cognition-enhancing effects of D-cycloserine in AD are controversial in other studies [113-117]. Another NMDAR modulator, GLYX-13, which is a monoclonal antibody-derived peptide acting as an agonist at NMDAR glycine binding site, can enhance learning and memory in both healthy young and impaired aging rats. Interestingly, the learning-improving effect is more prominent in old than in young rats [118, 119].

### Behavioral and Psychological Symptoms: the other Critical Therapeutic Dimension of Dementia

In addition to memory and cognitive function impairment, behavioral and psychological symptoms associated with dementia (BPSD) are main contributors to poor life quality of patients with dementia and burden to their caregivers. The concept of BPSD was developed in late 1980s and its definition was affirmed as “a term used to describe a heterogeneous range of psychological reactions, psychiatric symptoms, and behavior occurring in people with dementia of any etiology” in the International Psychogeriatric Association (IPA) consensus conference in 1996 [120]. Clinically, BPSD includes four domains: 1. behavior, including aggression, agitation, sleep disturbance, sexual disinhibition, etc. 2. mood, including depression, anxiety, mania, etc, 3. thought content, including delusions, illogical thoughts, etc, 4. perception, including hallucinations and misidentification [121-123]. The clinical manifestations of BPSD may vary with different types of dementia. For example, mood changes, such as anxiety, depression, emotional labiality and apathy, are more common and psychotic symptoms are fewer in vascular dementia (VaD) [124]. BPSD may be more severe in VaD than in AD [125]. Predominant symptoms of BPSD also differ at different stages of dementia. For example, depression is common in the early stage of dementia and psychotic symptoms occur more frequently with disease progression [126, 127].

The prevalence of BPSD is very high in patients with dementia, with 64% at initial evaluation [128] and 90% over the whole course of illness [129]. The severity of BPSD is related to the severity of dementia of all types [126, 130, 131]. The development of BPSD is associated with a poorer prognosis, a more rapid rate of cognitive decline and illness progression [132]. Besides, BPSD is distressful to patients, care givers, and professionals [133]. Untreated BPSD contributes to over-medication [132, 133], premature institutionalization [128, 129], increased financial cost [120], decreased quality of life for both the caregivers and the patients [134, 135], significant stress to caregiver [136], nursing staff in residential facilities [137], and excess disability [138].

### Lack of Effective and Safe Treatment for Behavioral Symptoms in BPSD

Although BPSD is very common in dementia and contributes to poor outcome, no pharmacological therapy is particularly effective [139] and no medication has yet been approved by the Food and Drug Administration (FDA) for treating BPSD to date.

Antipsychotic medications have been used clinically for treating the behavioral disturbances and psychosis of dementia, but the evidence of their efficacy and safety is scanty [140]. Although some studies of psychotropic agents reveal positive results, most of them are open label studies, or with small sample size [141]. Large-scale studies have demonstrated negative findings. For example, the Clinical Antipsychotic Trials of Intervention Effectiveness—Alzheimer’s Disease (CATIE-AD) indicates that the response rates of olanzapine, risperidone, and quetiapine are similar to that of placebo [142]. Moreover, atypical antipsychotics are associated with more deterioration of cognitive function in patients with AD [143], suggesting that antipsychotic medication is not a effective treatment option for both cognitive symptoms and BPSD.

In 2005, FDA (USA) issued a warning that, among elderly with dementia, treatment of behavioral disorders with atypical antipsychotics is associated with a higher mortality rate. Specific causes of deaths are mostly cardiac related or infections [144]. The FDA also suggests that typical antipsychotics have similarly higher mortality risk. This concern is confirmed by a meta-analysis showing that haloperidol, a widely used typical antipsychotic agent, carries a relative mortality risk of 2.07 [145] and by another study demonstrating that mortality risks with typical antipsychotics are higher than with atypical antipsychotic agents in elderly patients [146]. Another meta-analysis of 17 placebo controlled trials of atypical antipsychotics for the treatment of BPSD conducted by the FDA suggests a significant increase in mortality, too [145]. The Cochrane Reviews find that risperidone and olanzapine are useful in reducing BPSD, but both are associated with serious adverse cerebrovascular events and extrapyramidal syndrome. Thus, the Cochrane Reviews suggest that neither risperidone nor olanzapine should be used routinely to treat BPSD [147]. Taken together, these findings suggest that antipsychotic agents carry substantial risk for treating the dementia patients with BPSD.

The therapeutic effects of AChE inhibitors, which are the main medications for the treatment of AD, on the treatment of BPSD are uncertain. Although clinical trials evaluating behavioral effect of AChE inhibitors showed potential treatment benefit for behavioral symptoms, some randomized controlled studies revealed negative results (Table **1**). In a meta-analysis, there was small advantage of AChE inhibitors over placebo with a modest improvement of 1.72 points on the Neuropsychiatric Inventory (NPI) and 0.03 points on the Alzheimer Disease Assessment Scale, noncognitive (ADAS-noncog) [167].

Anticonvulsants, such as carbamazepine and valproate, are beneficial in the treatment of behavioral disturbance of dementia in small-scale studies; however, larger studies show negative results [132, 141, 168]. Therefore, anti-convulsants are not recommended for the treatment of BPSD [169]. 

In summary, due to the lack of effective and safe medication for treating behavioral disturbance in AD, novel therapeutic approach is desperately needed for the treatment of BPSD.

### The Therapeutic Potential of NMDA Modulators for the Treatment of Behavioral Symptoms in Alzheimer’s Disease

While BPSD may be an endophenotype of neurotransmitter-associated gene variation in AD [170], the etiology of BPSD is complex and unclear. Dysregulations in cholinergic, serotonergic, dopaminergic, noradrenergic, and γ-aminobutyric acid (GABA) neurotransmitter systems are all involved in the neurobiology of mood, thought and behavior disturbance in patients with dementia [120, 132, 171]. Considering the complexity and diversity of pathophysiology in different types of dementia, we focus the discussion on BPSD in AD. 

Briefly, decreased cholinergic function correlates with cognitive impairment and aggression in AD patients [172]. Deficits in cholinergic neurotransmission may also lead to behavioral disturbances such as psychosis, agitation and personality changes in AD [173]. Dysfunction of serotonergic system may contribute to BPSD in AD. Serotonin secretion and serotonin receptor density are decreased in AD patients [174]. Serotonin hypofunction may be linked to psychosis and anxiety and hyper-response of serotonin receptor may be associated with aggression in AD patients [174]. Genetic studies also revealed an association between serotonin neurotransmission and BPSD. Serotonin receptor 5HT2A polymorphism is associated with psychotic symptoms, such as hallucination and delusion, and aberrant motor behavior in AD [175]. Significant association was also found between genetic variation in the serotonin transporter gene and behavioral disturbances, such as aggression, irritability and psychosis in probable and mild AD patients [176-178].

Dopaminergic system dysfunction is related to AD patient with BPSD, especially the symptom of apathy [179]. A study indicates that the binding potential of dopamine receptor correlates inversely with severity of BPSD in AD [180]. Besides, there are correlations between dopamine associated gene polymorphism and BPSD, such as dopamine transporter gene 3' variable number tandem repeats (VNTR) and agitation, dopamine receptor 4 VNTR and mood component [178], and dopamine receptor 3 glycine allele and paranoid ideation [181].

Other neurotransmitter systems, such as norepinephrine (NE), GABA, are also related to BPSD. Loss of NE neuron with compensated increasing NE activity may lead to BPSD in AD [182]. Higher density of GABA receptors in the temporal cortex correlates with more severe depression in AD [183]. Another study suggests that plasma GABA concentration is related to depression and apathy in severe AD [184].

In addition, AD associated behavior disturbance may be partly due to aberrant NMDA neurotransmission. Although the definitive role of NMDA neurotransmission in BPSD still needs to be elucidated, NMDAR dysfunction is indeed involved in psychiatric and behavioral symptoms, such as agitation, depression, anxiety and psychotic symptoms. In animal models, NMDAR dysfunction is associated with agitation and aggression. NMDA neurotransmission is involved in regulating aggressive behavior in cat [185] and NMDAR hypofunction caused by NMDAR antagonist leads to behavior disturbance, agitation and aggression in rodent [186, 187]. Abnormal glutamatergic neurotransmission in the frontal and cingulate area is postulated to contribute to agitation in AD [188]. 

NMDAR dysfunction is also related to depression. Decreased expression of NR1 subunit is found in the prefrontal cortex of olfactory bulbectomized rat, which is a model of depression with anxiety [189]. NMDAR antagonists, such as ketamine, may have antidepressant effect [190, 191]. One possible underlying mechanism is that NMDAR antagonists activate mTOR signaling through α-amino-3-hydroxyl-5-methyl-4-isoxazole-propionate (AMPA), ERK and Akt pathways [190] and mTOR activation leads to increased spine density and synaptic activity in the prefrontal cortex of rats and produces antidepressant-like behavioral effect [190, 191]. 

NMDAR is involved in fear memory formation and extinction, which is the neural basis of anxiety disorder. NMDAR antagonists have anxiolytic effects both in rodents and humans [192]. Mice with reduced NMDAR-glycine affinity have less anxiety-like behaviors [193]. Besides, D-cycloserine, a partial agonist of NR1 subunit of NMDAR, can alleviate anxious symptoms in patients with anxiety disorder [194]. 

Dysfunction of NMDA neurotransmission also plays a critical role in the pathophysiology of schizophrenia, which presents with mood disturbance, psychosis and cognitive decline similar to dementia [27]. In fact, schizophrenia was first identified as dementia praecox, a term popularized by Emil Kraepelin. Clinical characteristics of BPSD include hallucinations, delusions, disorganized speech, and disturbing behavior resembled positive symptoms of schizophrenia; social withdrawal, apathy, alogia, and avolition resembled negative symptoms in schizophrenia, and behavior, sleep, or affect problems are also frequently encountered in schizophrenic patients. In genetic studies, the linkage analyses of AD with psychosis reveal some shared linkage alleles found in schizophrenia [195]. Animal studies also suggest that NMDAR antagonists result in anatomical and functional alterations in the same limbic structures presented in the dementia syndrome of schizophrenia and AD. This implies that NMDAR antagonists may serve as models for behavioral studies of both schizophrenia and dementia [196]. Taken together, these findings suggest that a partially shared susceptibility and pathophysiology may underline the psychiatric and behavioral disturbance in both schizophrenia and AD or dementia in general. 

Further analysis conducted by Maria found that a single nucleotide polymorphism (rs2153674) in the G72 (D-amino acid oxidase (DAAO) activator, DAOA) gene was associated with the occurrence of psychotic symptoms in patients with AD and this allele accounted for up to 15% of the variance in delusions severity [197]. This allele also has some linkage disequilibrium in schizophrenia [198]. DAOA is a primate-specific protein that enhances DAAO activity to degrade the potent neurotransmitter of glycine coagonist binding site in NMDAR, such as D-serine and D-alanine. Dysfunction of DAOA is associated with schizophrenia [198].

GSK pathway may play a key role in NMDAR dysfunction in BPSD. GSK pathway is associated with neuroprotection and mood disorder. Activated GSK pathway is involved in hyperdopamine receptor-mediated inhibition of NMDAR in the prefrontal cortex, which may contribute to the associated behavioral changes [199]. Consistently, suppressed GSK activity can reduce ketamine-induced psychotomimetic effects, such as locomotor hyperactivity, motor incoordination, sensorimotor impairment, and cognitive deficits [200]. 

Clinically, NMDAR dysfunction is found in AD patients with behavioral symptoms. In a study conducted by Tsang *et al*., twenty-one patients with AD were divided into high anxiety and low anxiety groups by Neuropsychiatric Inventory score and their NMDA subunits were assayed. They found that affinity of the glycine coagonist site of NMDAR was higher and the density of NR2A subunit was downregulated in AD patients with high anxiety score [201]. Above results imply that dysfunction of NMDA neurotransmission is related to behavioral and psychological symptoms of AD.

For the treatment of behavioral symptoms, memantine is postulated to have therapeutic benefits other than its modest efficacy for cognition, but the results are controversial. Significant benefits of memantine treatment on the measurement of behavior symptoms are observed in open label studies and as add-on dopenezil treatments (see Table **2**). However, in two randomized, double-blind, placebo-controlled trials comparing treatment effect of memantine to placebo, memantine is not better than placebo in improving the behavioral outcome [201, 204]. In another randomized, double-blind, placebo-controlled trials conducted by Fox* et al*., the findings are mixed. There is no significant difference between memantine and placebo in Cohen-Mansfield Agitation Inventory, but memantine has significant improvement over placebo in Neuropsychiatric Inventory [211]. Subsequent pooled analyses suggest that memantine is modestly effective in treatment of BPSD in domains of irritability, agitation/aggression and delusion [206, 207, 212]. However, caution needs to be taken when long-term attenuation of NMDAR function by memantine can contribute to the deterioration of mood and psychotic symptoms as other NMDAR antagonists do. 

Alternatively, NMDAR enhancers may improve the cognitive, mood and psychotic symptom in AD like in schizophrenia and major depression. Previous studies revealed that NMDAR enhancing agent can alleviate psychotic symptoms of patients with schizophrenia [213-217]. In addition, NMDAR-enhancing treatment can reduce depressive symptoms [213, 214, 218] and improve cognitive function in patients with schizophrenia [213, 214, 219, 220]. D-serine add-on therapy can improve positive symptoms, negative symptoms and cognitive function in treatment-refractory schizophrenia as well as 14.7% decrease of depressive symptoms in a 6-week trial [220]. In another 6-week trial of adjuvant glycine treatment of schizophrenia, glycine can lead to about 34% reduction in negative symptoms, 11% reduction in cognitive symptoms and small but significant improvements in depression and excitement subscales [219]. Sarcosine, a glycine transporter-I inhibitor which potentiates glycine's action on NMDAR glycine binding site, can result in a 17% reduction of the positive symptoms, a 14% reduction of the negative symptoms and 13% improvement in the PANSS-cognitive subscale after a 6-week add-on trial [213]. Recently, we also found that sarcosine can improve the symptoms of major depression with fast and high remission rate [221]. 

Agonism of the coagonist binding site of NMDAR may be another approach to improve NMDAR function for treatment of behavioral symptoms of AD. In addition to glycine, D-amino acids, especially D-serine, are the agonist of co-agonist binding site of NMDAR. D-serine can be derived from L-serine by serine racemace (SR) in astrocyte and be uptaked by alanine-serine-cysteine transporter (ASC-1) to pre-synaptic neuron and degraded by DAAO. Therefore, increase of synaptic D-serine level can be achieved by enhancement of SR, attenuation of ASC-1 or inhibition of DAAO [107]. DAAO inhibitor can also increase NMDAR associated synaptic activity without increasing D-serine level [222]. To our knowledge, there is no study conducted to explore the therapeutic effect of NMDAR enhancers in treatment of behavioral disturbance in AD. Due to their beneficial effects on cognition, mood, anxiety and psychosis in a variety of mental disorders, we predict there is a good likelihood that NMDAR enhancers can have therapeutic efficacy in behavioral and psychological symptoms of AD. This approach, once confirmed, will represent a novel treatment strategy for the behavioral disturbance in AD.

## Figures and Tables

**Table 1.** Behavioral Effect of AChE Inhibitors in Alzheimer’s Disease

Study	Design	Dosage (per day)	Duration	Result
*** Donepezil***				
Weiner, M. F., *et al*. 2000 [148]	Open-label	5-10mg	48weeks	Significant improvement in CBRSD^1^ total scores on Week 12. Significant improvement in CBRSD depression and behavioral dysregulation scores on Week 16. CBRSD scores returned to baseline levels on Week 48
Gauthier, S., *et al*. 2002 [149]	Double-blind, placebo-controlled	5-10mg	24weeks	Significant improvement in following items of NPI^2^ scores: depression/dysphoria, anxiety, and apathy/indifference.
Paleacu, D., *et al*. 2002 [150]	Open-label.	10 mg	24 weeks	Significant improvement in NPI scores
Bergman, J., *et al*. 2003 [151]	Add-on treatment. Randomized, open-label.	5mg	4 weeks	Significant improvement in PANSS^3^ scores.
Holmes, C., *et al*. 2004 [152]	Randomized withdrawal	5-10mg	12 weeks	Significant improvement in NPI scores in group of continued donepezil treatment.
Cummings, J. L., *et al*. 2006 [153]		5-10mg	20 weeks	No significant change of NPI score on Week 8 Significant improvement in NPI scores on Week 20.
Howard, R. J., *et al*. 2007 [154]	Randomized, Double blind.	10mg	12 weeks	No significant difference between donepezil and placebo in CMAI^4^ scores and NPI scores.
Pelosi, A. 2008 [155]	Randomized, double blind.	5-10mg	12 weeks	No significant difference between donepezil and placebo in CMAI agitation score and NPI scores.
Reekum, R. 2008 [156]	Randomized, placebo-controlled	5-10mg	12 weeks	No significant difference between donepezil and placebo in CMAI score
Carrasco, M. M., *et al*. 2011 [157]	Open-label, noncomparative, prospective	5-10mg	24 weeks	Significant improvement in NPI scores
*** Galantamine***				
Tariot, P. N., *et al*. 2000 [158]	Double blind, placebo controlled	8-24mg	20 weeks	Significant improvement in NPI scores of galantamine 16- and 24-mg/day groups compared to placebo.
Rockwood, K., *et al*. 2001 [159]	Randomized, double blind, placebo controlled	24-32mg	12 weeks	No significant difference in NPI scores
Monsch, A. U. and Giannakopoulos, P. 2004 [160]	Open-label.	8-24 mg	12 weeks	Significant improvements in NPI scores.
Herrmann, N., *et al*. 2005 [161]	Post hoc analysis of 3 randomized, double-blind, placebo-controlled trials.	16-32 mg	12-24 weeks	Significant improvement in total NPI scores and individual domains of agitation/aggression, anxiety, disinhibition, and aberrant motor behavior
*** Rivastigmine***				
Finkel, S. I. 2004 [162]	Meta-analysis of 3 randomized, placebo-controlled trials	6-12mg	24 weeks	Significant improvement in the items of behavioral component of CIBIC-plus5: paranoia, delusion, aggression, activity disturbance.
Cummings, J. L., *et al*. 2005 [163]	Prospective, open-label.	3-12mg	26-week,	Decreased NPI-NH^6^ score, but not statistically significant. Significant improvement of follow the items of NPI-NH scores: delusions, hallucinations, agitation, apathy/indifference, irritability/lability, aberrant motor behavior, nighttime disturbances, and appetite/eating changes
Edwards, K., *et al*. 2005 [164]	Open label	3-12mg	52 weeks	Significant improvements in following domains of NPI-NH: delusions, hallucinations, anxiety and euphoria
***Rivastigmine***				
Gauthier, S.,* et al*. 2007 [165]	Open label.	3-12mg	24 weeks	At the end point, 62.3%, 62.6% and 56.0% patients had improvements in symptoms of anxiety, apathy and agitation respectively. However, 6.8%, 7.7% and 6.8% patients had worse symptoms of anxiety, apathy and agitation respectively.
***Tarcrine***				
Kaufer, D. I., *et al*. 1996 [166]	Open label.			Improvement of NPI scores

1 CBRSD: CERAD Behavior Rating Scale for Dementia.

2 NPI: Neuropsychiatric Inventory

3 PANSS; Positive and Negative Symptoms Scale

4 CMAI: Cohen-Mansfield Agitation Inventory

5 CIBIC-plus: Clinician's Interview-Based Impression of Change Plus Caregiver Input scale

6 NPI-NH: Neuropsychiatric Inventory-Nursing Home

**Table 2.** Behavioral Effect of Memantine in Alzheimer’s Disease

Study	Design	Dosage (per day)	Duration	Result
Reisberg, B.,* et al*. 2003 [201]	Randomized, double blind, placebo controlled	20 mg	28weeks	No significant difference between memantine and placebo in NPI scores
Tariot, P. N., *et al*. 2004 [202]	Add-on dopenezil treatment. Randomized, placebo-controlled.	20 mg	24 weeks	Significant improvement in NPI total scores
Cummings, J. L., *et al*. 2006 [203]	Add-on dopenezil treatment. Randomized, double-blind, placebo-controlled,	20mg	24 weeks	Significant improvement over placebo in NPI^1^ total scores.
van Dyck, C. H., *et al*. 2007 [204]	Randomized, double blind, placebo controlled	20 mg	24 weeks	No significant difference between donepezil and placebo in NPI and BGP^2^ scores
Cretu, O., *et al*. 2008 [205]	Add-on dopenezil treatment. Randomized		24 weeks	Significant improvement in agitation/aggression compared with donepezil monotherapy
Gauthier, S., *et al*. 2008 [206]	Pooled analysis of 6 randomized, double blind, placebo controlled trials.	20mg	24 or 28 weeks	Significant improvement in NPI total scores and the items: delusions, hallucinations, agitation/aggression and irritability/lability
Wilcock, G. N., *et al*. 2008 [207]	Pooled analysis of 3 randomized trials	20mg	24 or 28 weeks	Significant advantage in following domains of NPI: agitation/aggression, delusion, hallucination.
Schmidt, R., *et al*. 2010 [208]	Naturalistic study		16 weeks	Improvement in NPI total scores.
Herrmann, N., *et al*. 2011 [209]	Open-label	20mg	12 weeks	Significant improvement in agitation/aggression domains of NPI-NH^3^.
Clerici, F., *et al*. 2012 [210]	Post-marketing surveillance study	20mg	6 months	Significant behavioral improvement in patients who taking 20 mg memantine daily.
Fox, C.,* et al*. 2012 [211]	Randomized, double blind, placebo controlled	20mg	12 weeks	No significant difference between memantine and placebo in CMAI^4^ Significant improvement over placebo in NPI total scores.

1 NPI: Neuropsychiatric Inventory.

2 BGP: Behavioral rating scale for geriatric patients.

3 NPI-NH: Neuropsychiatric Inventory-Nursing Home

4 CMAI: Cohen-Mansfield Agitation Inventory
